# Decreased colorectal cancer incidence and mortality in a diverse urban population with increased colonoscopy screening

**DOI:** 10.1186/s12889-021-11330-6

**Published:** 2021-06-30

**Authors:** Jennifer J. Brown, Charles K. Asumeng, David Greenwald, Matthew Weissman, Ann Zauber, Jared Striplin, Olivia Weng, Justin M. List, Shannon M. Farley, Sidney J. Winawer

**Affiliations:** 1 Bureau of Chronic Disease Prevention, NYC Department of Health and Mental Hygiene, New York, NY USA; 2 Mount Sinai Beth Israel/Icahn School of Medicine at Mount Sinai, New York, NY USA; 3 Memorial Sloan Kettering Cancer Center, New York, NY USA; 4 NYC Health + Hospitals, New York, NY USA

**Keywords:** Colorectal cancer, Colonoscopy, New York City, Disparities

## Abstract

**Background:**

Although colorectal cancer screening has contributed to decreased incidence and mortality, disparities are present by race/ethnicity. The Citywide Colon Cancer Control Coalition (C5) and NYC Department of Health and Mental Hygiene (DOHMH) promoted screening colonoscopy from 2003 on, and hypothesized future reductions in CRC incidence, mortality and racial/ethnic disparities.

**Methods:**

We assessed annual percent change (APC) in NYC CRC incidence, stage and mortality rates through 2016 in a longitudinal cross-sectional study of NY State Cancer Registry, NYC Vital Statistics, and NYC Community Health Survey (CHS) data. Linear regression tested associations between CRC mortality rates and risk factors.

**Results:**

Overall CRC incidence rates from 2000 decreased 2.8% yearly from 54.1 to 37.3/100,000 population in 2016, and mortality rates from 2003 decreased 2.9% yearly from 21.0 to 13.9 in 2016 at similar rates for all racial/ethnic groups. Local stage disease decreased overall with a transient increase from 2002 to 2007. In 2016, CRC incidence was higher among Blacks (42.5 per 100,000) than Whites (38.0), Latinos (31.7) and Asians (30.0). In 2016, Blacks had higher mortality rates (17.9), than Whites (15.2), Latinos (10.4) and Asians (8.8). In 2016, colonoscopy rates among Blacks were 72.2%, Latinos 71.1%, Whites 67.2%, and Asians, 60.9%. CRC mortality rates varied by neighborhood and were independently associated with Black race, CRC risk factors and access to care.

**Conclusions:**

In a diverse urban population, a citywide campaign to increase screening colonoscopy was associated with decreased incidence and mortality among all ethnic/racial groups. Higher CRC burden among the Black population demonstrate more interventions are needed to improve equity.

## Introduction

Colorectal cancer (CRC) is the third most common cancer and second leading cause of cancer death for men and women in the U.S. [1] Incidence rates fell 2.4% yearly, as did mortality rates, 2.2% yearly over 2007–2016 [2]. Early detection and prevention have contributed to decreases in CRC incidence and mortality. Reductions have been associated with screening by colonoscopy, sigmoidoscopy, guaiac-based fecal occult-blood testing (FOBT), and fecal immunochemical tests (FIT) [3–11]. Colonoscopic polypectomy has reduced CRC mortality by reducing CRC, [12, 13] and FOBT has reduced long-term CRC mortality [14, 15], primarily by detection of early-stage CRC. A community-based screening program in California, utilizing annual FIT and colonoscopy, achieved greater than 80% participation, with associated reductions in CRC incidence, mortality and advanced stage disease [16]. A population-based FIT screening program in Italy was associated with lower mortality rates, while incidence peaked during the introduction of the program and then returned to baseline [9].

New York City (NYC) differs from prior studies as a large and diverse urban population. It has an average of 3500 new CRC cases and 1200 deaths annually [17]. The NYC DOHMH convened C5 in 2003 to increase CRC awareness and screening by colonoscopy. The goals were to increase screening for CRC and adenomatous polyps in NYC adults ages 50 and older, eliminate racial and ethnic screening disparities, and reduce CRC incidence and mortality [18].

Previously, we reported increased colonoscopy rates in NYC from 41.7 to 61.7% from 2003 to 2007 as well as a reduction in disparities between racial and ethnic groups by 2010, and a further increase in screening rates to 69% in 2013 [18, 19].

The specific aim of our study, reported in this paper, was to determine the impact on CRC incidence and mortality of the screening program which used primarily colonoscopy and was targeted to the diverse NYC population. Our hypothesis was that the reduction in screening disparity in our various ethnic population groups was associated with comparable rates of incidence and mortality reduction in these groups, with a transient incidence increase. These observations would help clarify the impact of screening access on the outcome of CRC in diverse groups.

## Methods

### Institutional review board (IRB) approval

Study methods for the NYC Community Health Survey (CHS) data used were approved by the NYC Health Department’s IRB. The New York State Cancer Registry and NYC Vital Statistics data are publicly available for use in secondary analysis; therefore, IRB approval was not required.

### CRC screening interventions

Citywide initiatives from 2003 to 2016 to achieve increased rates of screening by colonoscopy are previously described [18] and included public education, healthcare professional education, public health detailing in areas of higher CRC mortality and larger proportion of Black race, patient navigator programs for colonoscopy at public and voluntary hospitals [20], a direct referral initiative from primary care to colonoscopy, a NYC colonoscopy quality initiative, free colonoscopy for the uninsured at community health centers and participating endoscopy centers, and NYC-specific practice recommendations for screening by colonoscopy as a City Health Information bulletin [21].

### CRC age-adjusted incidence and mortality data sources

CRC incidence data including stage of disease at diagnosis for NYC residents were obtained from the New York State Cancer Registry for 1975 to 2016, *n* = 173,388. Incidence by race/ethnicity and borough of residence were obtained from the NY State Cancer Registry for 2000 to 2016, *n* = 65,550 [17]. CRC mortality data by race/ethnicity, borough of residence, and NYC United Hospital Fund neighborhood were obtained from the NYC Office of Vital Statistics for 2003 to 2016, derived from death certificates, *n* = 19,241 [22]. For neighborhood estimates, 2012 to 2016 data were pooled. All data were age-adjusted. White, Black, and Asian/Pacific Islander (Asian) race/ethnicity groups included only non-Latinos. Linear regression tested associations between CRC mortality rates, race/ethnicity, CRC risk factors and access to care identified in the NYC CHS.

ArcGIS Desktop 10.6.1 was used to map CRC age-adjusted mortality rate data from 2014 to 2016 as against black population in 42 designated United Health Fund (UHF) areas of NYC.

### CRC screening data sources

This study included colonoscopy status of NYC residents ages 50 and older who responded to the NYC CHS 2003 to 2016 (*N* = 9000 surveyed annually as representative of NYC) [23]. The survey is a population-based, representative study of NYC residents. Based on the CDC’s Behavioral Risk Factor Surveillance System (BRFSS), CHS is a random-digit-dial telephone survey conducted annually since 2002. CHS uses a dual frame sample design consisting of random-digit-dial landline telephone exchanges and a second frame of cellular telephone exchanges that cover NYC. CHS also incorporates a disproportionate stratified random sample design. Data from 14 CHS cycles were used in the current analysis (2003–2016).

The survey includes sociodemographic and health behavior questions including colonoscopy, smoking, having fruits and vegetables in the diet, and access to healthcare. In two years of the study, 2003 and 2012, a question about timely stool-based CRC screening was included in CHS, but this question was not included in the CHS the other years of the study (2002, 2004–2011,2013–2016). We limited our analyses to ages 50 and older because the NYC screening recommendations advised starting at age 50 without an upper age limit. The United States Preventive Services Task Force recommended colonoscopy screening every 10 years for average-risk people ages 50–75, with individualized screening for ages 76–85 [24]. Respondents who reported a colonoscopy in the past 10 years, including both screening and diagnostic, were considered to have received timely colonoscopy and are included in this analysis. Data on colonoscopy by race/ethnicity was not available prior to 2003. We examined additional CHS variables including borough of residence and for some analyses, 2012–2016 data were combined to show significant differences.

### Statistical analyses

The Joinpoint Regression Program (National Cancer Institute, version 4.5.0.1) modeled CRC incidence, mortality and screening curves by race/ethnicity from the underlying rates (age-adjusted to the 2000 U.S. Standard Population). Annual percent change (APC) and annual average percent change (AAPC) were considered statistically significant at *P* < 0.05 using a two-sided test [25]. For all analyses presented, APC did not differ from AAPC, therefore we only report the APC. APC was tested for parallelism to identify differences in trends for regression mean functions among pairs of race/ethnicities. Racial/ethnic comparison trends of incidence overall, mortality overall, and its relative associations with screening rates overall were analyzed using SAS Version 9.4. The covariate (screening rates) is included in the model for determining the effect of trends of incidence and mortality. ArcGIS was used to map CRC mortality and Black population by United Hospital Fund (UHF) neighborhood.

All authors had access to the study data and reviewed and approved the final manuscript.

## Results

### NYC CRC incidence and stage at diagnosis trends

**Fig. 1:**
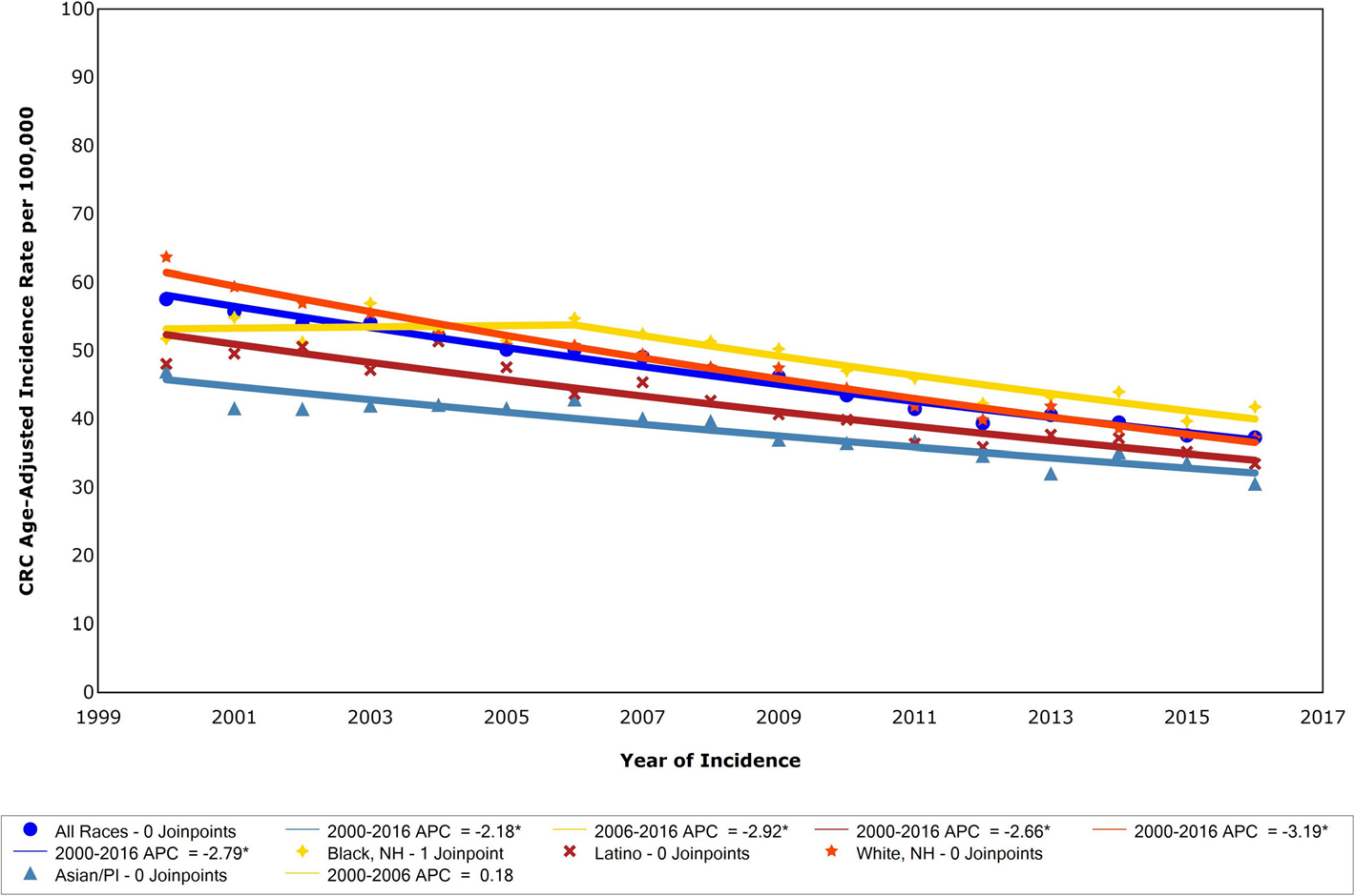
Joinpoint assessed APC in CRC incidence in NYC among all races, Asian, Black, Hispanic, and White adults, 2000–2016. APC = annual percent change, CRC = colorectal cancer, NYC=New York City

Age-adjusted incidence of cases for all CRC stages combined in NYC declined significantly from 2000 to 2016, from 57.5 to 37.3 per 100,000 population, (APC = − 2.79, *p* < 0.0001; *n* = 65,550), Fig. 1. Decrease in incidence rates by borough was significant and similar: Bronx APC = − 3.3; Brooklyn APC = − 3.1; Manhattan APC = − 3.6; Queens APC = − 2.5; and Staten Island APC = − 2.7. A test for parallelism between boroughs did not show significant differences. In 2016, CRC incidence per 100,000 was significantly higher among Blacks, at 42.5 (95% CI: 39.7–45.4) than Whites 38.0 (95% CI: 35.9–40.1, *p* = 0.01), Latinos 31.7 (95% CI: 29.4–34.1, *p* < 0.0001) and Asians 30.0 (95% CI: 27.2–33.2, p < 0.0001). The decrease in CRC incidence rate was similar and significant for each group: White APC = − 3.19; Latino APC = − 2.66; Black APC = 0.18 for 2000–2006 and APC = − 2.92 for 2006 to 2016; and Asian APC = − 2.18, respectively. Comparison between race/ethnicities showed rates of decline were highest among White and lowest among Asian residents. The incidence by stage of diagnosis trend from 1976 to 2016 decreased over time overall and for regional disease during the screening campaign, but local stage disease increased from 2002 to 2007 at 4.26% annually, and then decreased at 1.17% annually from 2007 to 2016, Fig. 2**.** A transient increase in local CRC at diagnosis would be expected with increasing colonoscopy, as it allows identification of the disease at an earlier stage.

**Fig. 2:**
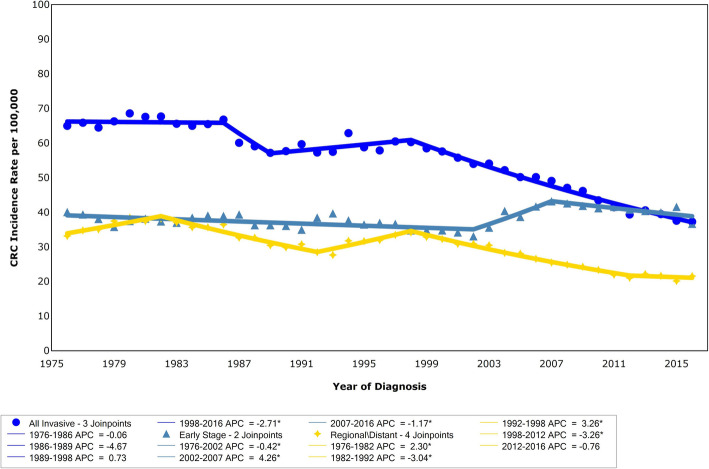
Overall incidence by stage of CRC: all invasive cases, early stage, or regional stage, 1975–2016. Data are from the New York State Cancer Registry. ^ Indicates a significant trend. APC = annual percent change, CRC = colorectal cancer, NYC=New York City

### NYC CRC mortality trend

**Fig. 3:**
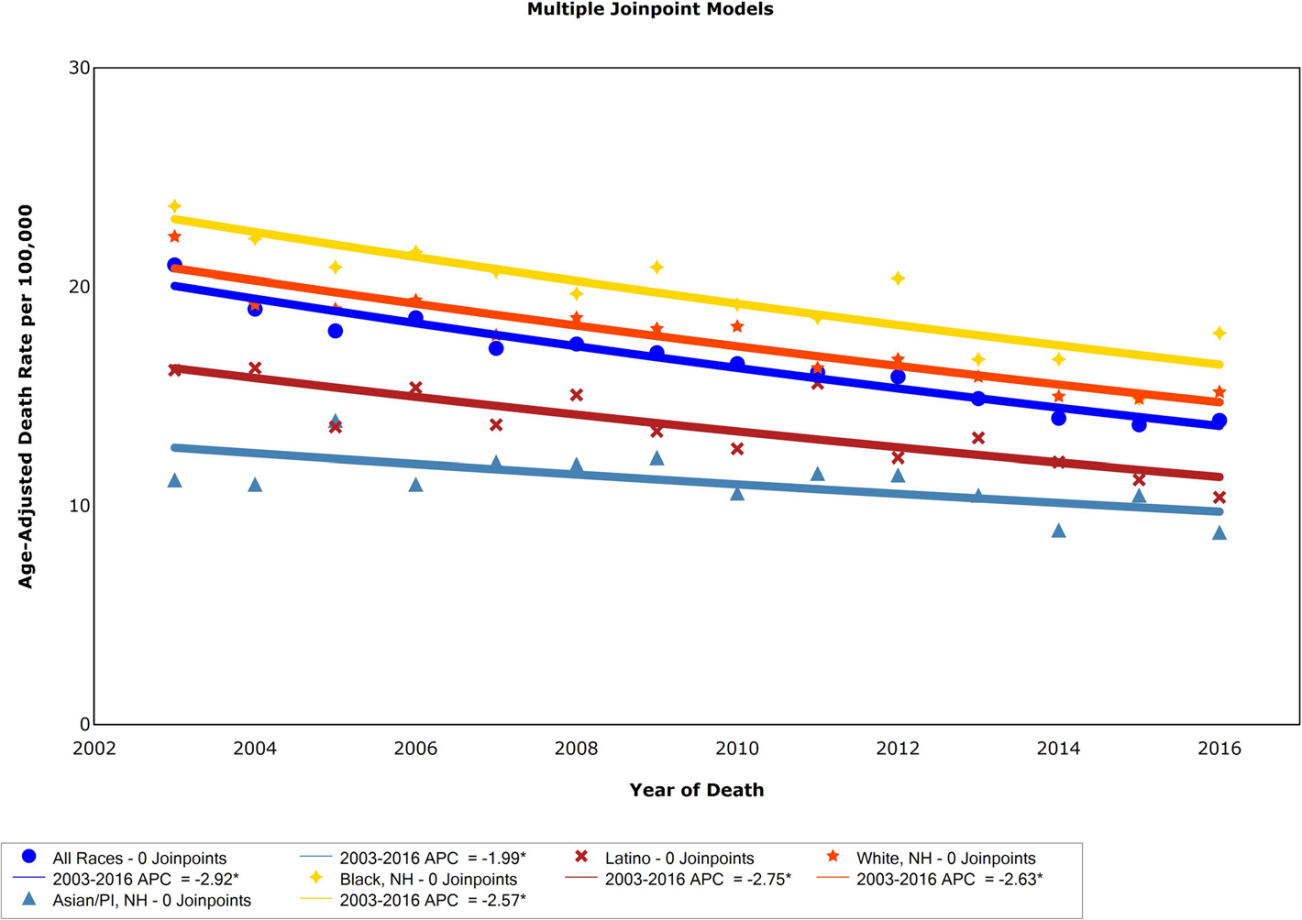
Joinpoint assessed APC in CRC mortality among all races, Asian, Black, Hispanic, and White adults. Data are from NYC Vital Statistics, 2003–2016. APC = annual percent change, CRC = colorectal cancer, NYC=New York City. ^ Indicates a significant trend

Overall, the CRC age-adjusted mortality rate decreased significantly from 2003 to 2016, from 21.0 to 13.9 per 100,000 NYC population with an APC of − 2.92 (*p* < 0.0001; *n* = 19,241), Fig. 3. CRC mortality decreased significantly over time similarly for each group (Black APC = − 2.57; White APC = − 2.63; Latino APC = − 2.75; and Asian APC = − 1.99, respectively). Comparisons between race/ethnicities showed the APC values for Blacks and Whites were not significantly different, while the APC for Latinos was higher and that of Asians was lower. In 2016 citywide, significantly higher CRC mortality rates per 100,000 were experienced by Blacks with 17.9 deaths (95% CI: 16.1–19.7) than by Whites with 15.2 deaths (95% CI: 13.9–16.4, *p* = 0.01) Latinos 10.4 (95% CI: 9.0–11.8, *p* < 0.0001) and Asians 8.8 (95% CI: 7.1–10.4, *p* < 0.0001).

Decrease in CRC mortality rates by borough were significant: Bronx APC = − 3.1; Brooklyn APC = − 3.3; Manhattan APC = − 3.4; Queens APC = − 2.5; Staten Island APC = − 2.5. Comparisons between boroughs showed the rates were not significantly different. CRC mortality rates in 2016 for New Yorkers living in Staten Island were 14.5 (95% CI: 11.6–18.0), the Bronx 14.0 (95% CI: 12.0–15.9), and Brooklyn 13.3 (95% CI: 11.9–14.6). The rates of CRC mortality in Staten Island and the Bronx were significantly higher than those in Queens at 11.5 (95% CI: 10.2–12.7) and Manhattan at 11.5 (95% CI: 10.0–13.0). CRC age-adjusted mortality rates varied by neighborhood across NYC, for pooled data of 2014–2016, Fig. 4. Areas of higher CRC mortality rates shown by darker shading showed an association with areas of a higher proportion of Black New Yorkers in the community, shown by larger circles. CRC mortality was not associated with the proportion of Whites, Latinos or Asians (not shown).

**Fig. 4:**
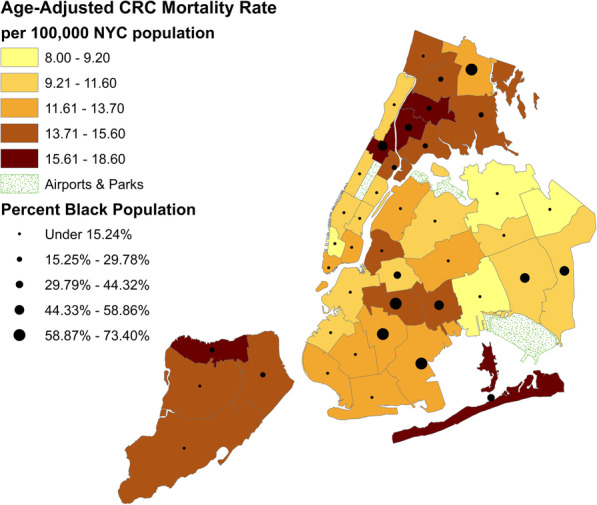
CRC mortality and percent Black population map of NYC. Mortality rate is from NYC Vital Statistics 2014–2016. Map is the authors’ own. ArcGIS Desktop 10.6.1 was used to map CRC age-adjusted mortality rate data from 2014 to 2016 as against black population in 42 designated United Health Fund (UHF) areas of NYC. CRC = colorectal cancer, NYC=New York City

Higher rates of CRC mortality were significantly and independently associated with Black race compared with others (*p* < =.0001), lacking fruits and vegetable in the diet (*p* < =.0001), and with a higher proportion of people who smoked (*p* = 0.0026), had no health insurance (*p* < =.0001), and had no primary care provider (PCP) as a usual source of health care (*p* = .0254), Table 1.

**Table 1:** CRC Cancer Mortality Rate per 100,000 NYC Population by CRC Risk Factor and Access to Healthcare

CRC Risk factor	Colon cancer mortality rate per 100,000	95% CI	***P***-value
**Race/ethnicity**			< 0.0001
Black	15.18	14.20–16.16	
White	12.02	11.38–12.65	
Latino/a	10.27	9.46–11.08	
Asian	8.84	7.84–9.85	
**Diet**			
Consumed fruits/vegetables	13.17	13.14–13.20	<=0.0001
No fruits/vegetables	13.41	13.32–13.50	
**Smoking**			
Never smoked	13.17	13.14–13.21	<=0.0026
Currently smoke	13.42	13.34–13.50	
**Access to healthcare**			
Have a PCP	13.19	13.16–13.22	<=0.0254
Don’t have a PCP	13.28	13.20–13.36	
**Access to health insurance**			
Have health insurance	12.96	12.88–13.05	<=0.0001
Uninsured	13.25	13.22–13.28	

Legend: Mortality rate data are from NYC Vital Statistics 2014–2016. Risk factor and access to healthcare are from responses to CHS questions in 2014–2016 on diet (About how many cups of fruit did you eat yesterday? About how many cups of vegetables did you eat yesterday?), smoking (Have you smoked at least 100 cigarettes in your entire life? Do you now smoke cigarettes every day, some days, or not at all?), access to healthcare (Do you have one or more person you think of as your personal doctor or health care provider? Do you have any kind of health insurance coverage, including private health insurance or governmental plans such as Medicare or Medicaid?) *CRC* colorectal cancer, *NYC* New York City, *PCP* primary care provider, *CHS* community health survey

### NYC colonoscopy trends

**Fig. 5:**
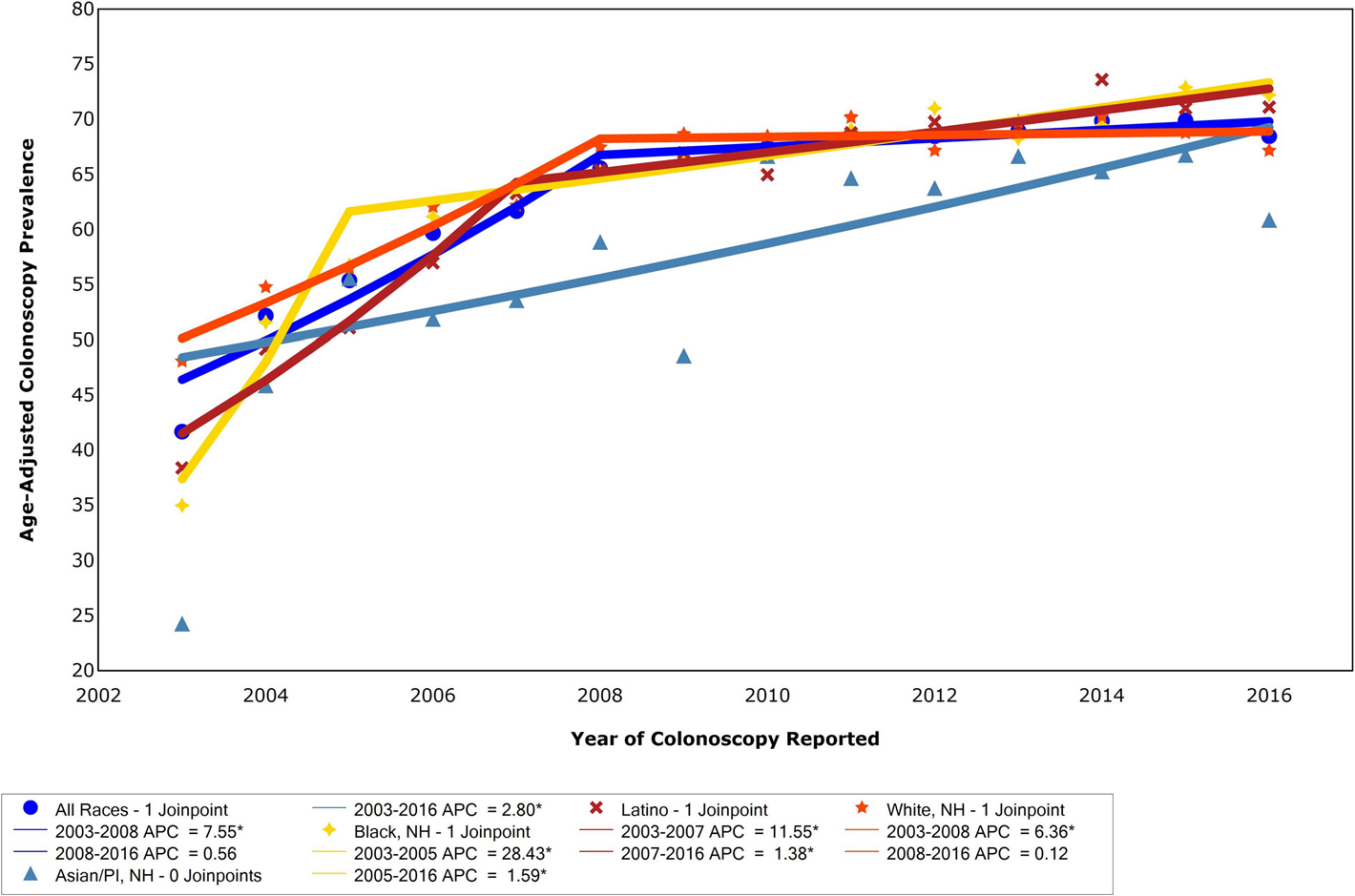
Age-adjusted timely colonoscopy was defined as a colonoscopy within the last 10 years, from CHS questions (A colonoscopy is an exam in which a tube is inserted in the rectum to view the bowels for signs of cancer or other health problems. Have you ever had a colonoscopy? When was your most recent colonoscopy?), 2003–2016. Joinpoint assessed APC in timely colonoscopy among all races, Asian, Black, Hispanic, and White adults 50 years and older. APC = annual percent change, NYC=New York City, CHS = community health survey. ^ Indicates a significant trend

From 2003 to 2016, timely colonoscopy increased in NYC overall from a starting point of 41.7 to 68.5%, Fig. 5. In NYC overall in 2016, more than 1.6 million NYC residents ages 50 and older had colonoscopy within the past 10 years. Modeling found two segments in overall timely colonoscopy: an increase from 2003 to 2008, APC = 7.55, and from 2008 to 2016 APC = 0.56. In 2016, Blacks had the highest screening rate, 72.2%. Asians had the lowest screening rate, 60.9%, significantly lower than Blacks (*p* = 0.0045) and Latinos (71.1%, *p* = 0.0092), while not significantly different from Whites (67.2%, *p* = 0.0885). An initial increase in timely colonoscopy was most rapid among Blacks (APC = 28.43) from 2003 to 2007. Increase among Latinos was APC = 11.55 from 2003 to 2007, and among Whites APC = 6.36 from 2003 to 2008, all significant. The Asian increase was consistent (APC = 2.92) and significant for the span of 2003 to 2016. Increase in timely colonoscopy was more gradual in recent years among the other race/ethnicities.

The colonoscopy rate in the eligible population was not significantly different among boroughs in 2016: Manhattan 71.9%, Bronx 70.8%, Staten Island 62.4%, Queens 67.4%, and Brooklyn 67.2%.

Data on timely stool-based CRC screening in NYC were not available for the same time period, but at only two points, in years 2003 and 2012. Stool-based testing is less often used in NYC, and the question is not asked every year on the CHS. In 2003 screening increased from 11.9% to 53,6% including both stool-based testing and colonoscopy. In 2012 screening increased 1.6% from 68.5% colonoscopy only, to 70.1% including both methods of testing. This indicates that use of stool-based testing was decreasing in NYC at a time when colonoscopy was increasing.

#### Association of CRC incidence/mortality and screening rate

**Fig. 6:**
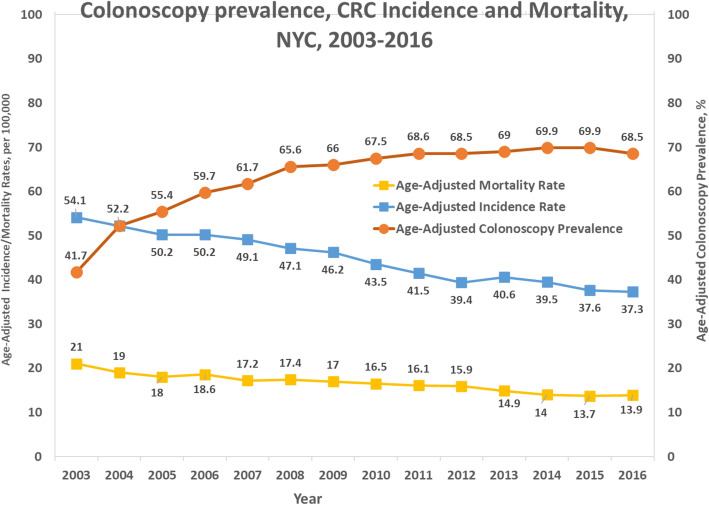
Age-adjusted timely colonoscopy in relation with CRC incidence and mortality rates, 2003–2016. Incidence data are from the New York State Cancer Registry. Mortality data are from NYC Vital Statistics. Colonoscopy data were from CHS. CRC = colorectal cancer, CHS=NYC Community Health Survey

NYC Colorectal cancer screening rate increased by 64.3%, from 41.7% in 2003 to 68.5% in 2016. In the same period, CRC age-adjusted incidence and mortality rates decreasing trends were highly significant with increasing screening (*p* = 0.0091). Figure 6 depicts overall trends of incidence and mortality over colonoscopy prevalence from 2003 to 2016. All individual races have similar graphs (not shown).

## Discussion

Between 2000 and 2016, CRC age-adjusted incidence and mortality rates declined in NYC. Local stage disease incidence increased between 2002 and 2007 during the time of rapidly increasing colonoscopy rates and decreasing stool-based testing, then decreased thereafter. This is expected because colonoscopy identifies early stage CRC. Overall incidence decreased by 2.8% annually and mortality by 2.9% annually, higher than national trends of a 2.4% yearly reduction in incidence, and 2.2% yearly reduction in mortality [2]. Given the diversity of the NYC population (32.1% White, 29.1% Latino, 24.3% Black, and 14% Asian) [26], the average percent decreases in mortality over time were not different between White and Black groups. National data from 1975 to 2012 showed CRC incidence declined more for Whites than Blacks, by 1.4% per year and 0.5% per year, respectively [27]. The reasons for this trend are not clear but require investigation. Explanations from other studies include less screening [28] and that Blacks were more likely to be diagnosed with advanced CRC than whites, limiting treatment options and contributing to poor survival and mortality [27, 29]. The CRC outcomes in Blacks have been variously attributed to multiple factors such as a genetic predisposition to CRC, a higher prevalence of CRC risk factors, and structural racism including differences in access to preventive and healthcare services such as cancer screening, and to timely and appropriate treatment [27, 29].

In our study, CRC mortality rates varied by NYC neighborhood where higher mortality was independently associated with both race/ethnicity as the proportion of Blacks in the population and known CRC risk factors such as: smoking, and lack of fruits and vegetables in the diet. Higher CRC mortality was also independently associated with barriers in access to health care such as: lack of health insurance and lack of a PCP. Although these variables were associated with higher mortality, it is not clear to what extent they may have been causal.

Disparities were found as CRC incidence rate was 11% higher among Blacks, compared with Whites; mortality was 18% higher among Blacks compared with Whites. The causes of these racial inequities are not clear. Overall CRC incidence in 2016 was similar in the US and NYC (37.5 vs, 37.3 per 100,000, US v. NYC [17, 26]). However, national data from 2007 to 2014 U.S. SEER demonstrated nearly double the disparities in mortality with Blacks having a 32% higher risk of CRC death compared to Whites [28]. Further, U.S. data from the CONCORD-2 study demonstrated increased CRC burden among Blacks where survival was significantly lower and had not reached the level among Whites diagnosed 15–20 years earlier [29]. This demonstrates a need for further study of the causes of CRC survival inequities in the Black population.

During this same time period of a decline in CRC incidence and mortality, NYC observed an associated 64% increase in the proportion of adults ages 50 and older reporting timely colonoscopy after a citywide campaign by C5 and DOHMH, reaching 68.5% in 2016. This increase occurred when NYC DOHMH promoted colonoscopy as the preferred CRC screening method, and when it was the only screening test consistently tracked by DOHMH surveys [23]. NYC’s rate of timely colonoscopy may be an underestimate of CRC screening from all tests, as it does not include screening by stool-based tests [30]. Stool-based tests are less often used in NYC than colonoscopy for CRC screening, and were not promoted during the campaign except for cases where a patient was unwilling or unable to get a colonoscopy. We found the age-adjusted timely colonoscopy rates among people ages 50 and older living in each of the boroughs of NYC were not significantly different, using pooled data from 5 years (2012–2016). Another NYC study of data from 2014, found that people living in other boroughs were less likely to be screened for CRC by colonoscopy than those living in Manhattan [31].

### CRC screening in other studies

Other studies in different populations have observed reductions in CRC incidence and mortality associated with increased CRC screening. These studies did not have the diversity present in our study. A case control study of mostly men from the Veterans Affairs healthcare system found that colonoscopy, including both screening and diagnostic procedures, was associated with a 61% reduction in CRC mortality [4]. A regional FIT screening program in Italy was associated with a 22% reduction in CRC mortality, and similar to our results, found that incidence rate peaked during the introduction of the screening program [9]. Compared with NYC’s 31% reduction in CRC incidence and 34% reduction in mortality reported here, a community-based Kaiser Permanente study in Northern California found a 25.5% reduction in CRC incidence and 52.4% reduction in CRC mortality. This was associated with an organized screening outreach program that increased the eligible population screened from 38.9% in 2000 to 82.7% in 2015 [16]. Program screening was by a mix of tests, primarily by fecal test and colonoscopy, and to a lesser extent sigmoidoscopy. Prior to our analysis, we did not anticipate a similarly large impact with primarily colonoscopy across an entire diverse urban center such as NYC.

### Disparities in CRC screening

In NYC, the uptake of timely colonoscopy as a screening method was initially rapid from 2003 to 2008, but subsequent periods tapered off to a plateau. Increase in colonoscopy was most rapid among Black New Yorkers, which could be expected as the interventions promoting colonoscopy such as public health detailing were more intensive in NYC areas of higher CRC mortality and a higher proportion of Black race [18]. However, our recent data through 2016 show that Asians had a lower colonoscopy rate overall compared to Black and Latino New Yorkers. Asian communities were not prioritized for the NYC colonoscopy promotion campaign due to the relatively lower risk of CRC in this population. Lower screening rates among Asians is consistent with findings from California and suggests a need for interventions about CRC prevention targeted to Asians [32]. In addition, offering patients the choice of colonoscopy or other CRC screening tests, for example stool-based tests, may increase screening beyond the current plateau, as some patients are unwilling to have a colonoscopy [33].

Nationwide public health programs to increase CRC screening such as the National Colorectal Cancer Roundtable (NCCRT) 80% by 2018 campaign (now named 80% in Every Community) may have contributed to lowering CRC burden nationally as well as in NYC, in addition to the C5 and DOHMH campaign from 2003 to 2016 [34]. Implementation of the Affordable Care Act and updated national clinical screening guidelines are trends during the specified dates which could have also increased colonoscopy screening rates.

As a strategy, screening at a younger age may benefit at-risk groups that disproportionately carry the burden of CRC at younger ages [28]. Adding fecal testing methods to the recommended screening colonoscopy in NYC could potentially further increase rates by encouraging shared decision making and considering patient preference for the type of test [35]. These changes were incorporated into the latest C5 and Health Department NYC CRC screening recommendations in March 2020 to promote screening beginning at age 45, and individuals at familial or other increased risk before age 45, with a choice of colonoscopy or stool-based test as the screening test [36].

### Limitations

This is an ecological analysis where trends from three different sources are examined but are not based on individuals with paired data from the incidence or mortality status and the CRC screening status. Data from NYC included ages 50 and older, whereas national data were for ages 50 to 75. Unlike prior reports, citywide NYC data are not from a closed system, which adds associated limitations of inability to share patients’ health records and screening data among providers. Limitations include lack of CRC incidence and mortality by race ethnicity in the data sources prior to 2003. Also, screening modalities other than colonoscopy such as fecal testing were not consistently available in CHS survey data. Strengths of this study include its large scale, focus on colonoscopy, and data sources for a diverse population, which may be generalizable to other diverse urban environments.

## Conclusions

From 2003 to 2016, timely colonoscopy rates in NYC increased after a multifaceted citywide public health campaign by the DOHMH and a coalition of stakeholders, C5 [18]. During this time a decreasing burden of CRC disease was evident from significantly declining incidence and mortality rates for Black, White, Latino and Asian groups, following an initial transient increase in overall incidence of local stage disease. While some racial and ethnic disparities in screening were reduced, lower screening rates among Asians demonstrate the need for continued efforts in CRC prevention. Higher CRC burden among the Black population demonstrate a need to examine the causes and improve equity. This remains a top priority in order to further decrease the burden of CRC in all racial and ethnic groups.

## Data Availability

Supporting data are available from the author upon reasonable request. Data sources include the New York State Cancer Registry. Available at: https://www.health.ny.gov/statistics/cancer/registry/vol1/v1rnyc.htm; New York City Vital Statistics. Available at: https://www1.nyc.gov/site/doh/data/data-sets/vital-statistics-data.page; and NYC Department of Health and Mental Hygiene (DOHMH) Community Health Survey, 2003–2015. Available at: https://www1.nyc.gov/site/doh/data/data-sets/community-health-survey.page

## Abbreviations

NYC: New York City
CRC: colorectal cancer
C5: Citywide Colon Cancer Control Coalition
DOHMH: NYC Department of Health and Mental Hygiene
APC: annual percent change
AAPC: annual average percent change
CHS: NYC Community Health Survey
FOBT: guaiac-based fecal occult-blood testing
FIT: fecal immunochemical tests
PCP: primary care provider
NCCRT: National Colorectal Cancer Roundtable
